# *In vitro* Comparison of Apical Debris Extrusion Using Rotary and Reciprocating Systems in Severely Curved Root Canals

**DOI:** 10.22037/iej.2017.07

**Published:** 2017

**Authors:** Abbas Delvarani, Nahid Mohammadzadeh Akhlaghi, Raana Aminirad, Sohrab Tour Savadkouhi, Seyed Aliakbar Vahdati

**Affiliations:** a *Dental Material Research Center, Dental Branch, Azad University, Tehran, Iran; *; b * Department of Endodontics, Cranio-maxillo-facial Research Center, Dental Branch, Islamic Azad University, Tehran, Iran; *; c *Private Practice, Mianeh, East Azerbaijan, Iran; *; d *Department of Endodontics, Dental School, Loma Linda University, CA, USA*

**Keywords:** Apical Extrusion, Curved Canals, Reciprocating File, Root Canal Preparation, Rotary Instrumentation

## Abstract

**Introduction:**

The aim of this *in vitro* study was to compare the amount of apically extruded debris after root canal preparation using rotary and reciprocating systems in severely curved root canals.

**Methods and Materials:**

Thirty six extracted human mandibular first molars with 25-35^°^ curvature in their mesiobuccal (MB) canal (according to Schneider’s method) were cleaned and shaped with ProTaper and WaveOne systems. The extruded debris was collected and their net weight was calculated. To compare the efficiency of the two systems, the operation time was also measured. The data were analyzed with t-test.

**Results:**

The amount of extruded debris in WaveOne group was significantly greater in comparison with ProTaper group (26%). The operating time for ProTaper was however, significantly longer than WaveOne.

**Conclusion:**

Both root preparation systems caused some degree of debris extrusion through the apical foramen. However, this amount was greater in WaveOne instruments.

## Introduction

It is well documented that upon root canal treatment, and during cleaning and shaping of the canals, vital and necrotic tissues, microorganisms, dentinal debris and irrigation solutions extrude from the apical foramen [1-5]. These extrusions are one of the well-known key contributors to flare-ups as a result of periradicular inflammation, pain and swelling [6].

Studies have shown that different preparation techniques, such as step-back, and crown-down and different filing motions such as pull-push, result in different amount of debris extrusion (DE) [7-9]. Even though the introduction of the rotary systems facilitated and accelerated the root canal procedures, DE into the periapical space continues to occur [9]. Different studies evaluated the amount of extruded debris after using various engine-driven systems with different movements [10-19]. The comparison of hand and engine-driven filing systems also indicated that despite the advancement of the instruments and irrigation systems, and changes in the material, shape, pitch, taper and the motion cycle, all preparation techniques and instruments are associated with some amount of DE [20-24]. In the recent years, faster mechanical preparations, with a reduced number of instruments, led to the increased popularity of the single-file systems; nonetheless, it is a hypothesis that the significant amounts of dentin cutting in relatively shorter time periods may result in forcing more debris and irrigants through the apex [19]. Additionally reciprocation motion, as the mechanism of action in most of the single-instrument systems, mimics the kinematics of balanced force technique, which is proven to be a pressure less movement pushing less material in apical direction [25-27]. However, since the reciprocation is presumed to be a forceful movement, it may pump debris and irrigants through the apex like a mechanical piston [19].

Clinically, in multi-rooted posterior teeth, the practitioner is frequently faced with severely curved roots [27]. Studies showed contradictory results comparing rotary and reciprocating systems in single and multi-rooted teeth with mild to moderate curvature [1, 3, 19, 22, 23]. However, to date no study assessed the amount of extruded debris in severely curved canals between the full-sequence rotaries and reciprocating motion systems in multi rooted posterior teeth. The present study aimed to compare the ProTaper Dentsply Maillefer, Ballaigues, Switzerland) and WaveOne (Dentsply Maillefer, Ballaigues, Switzerland) systems for this particular aspect. 

ProTaper files (Dentsply Maillefer, Ballaigues, Switzerland) develop a “progressive preparation” in both vertical and horizontal directions with the progressively variable tapers of each instrument except for the F2 (25.08), F3 (30.09) and F4 (40.06) instruments which only have progressive taper only in the first 3 mm of the instrument and a decreasing taper thereafter up to final portion of the active part. The file cross-sectional design is very similar to a reamer, with a convex core and three machined cutting edges [22, 23, 28].

WaveOne (Dentsply Maillefer, Ballaigues, Switzerland) is a single-file NiTi system with reciprocating back-and-forth movement and a reverse taper, variable helical angle and a non-active edge. It is used with 150^° ^counter clockwise rotation (direction of cutting) and 50^° ^clockwise rotations at a speed of 300 rpm [29]. WaveOne is available in different tip sizes and tapers 21/0.06 (Small), 25/0.08 (Primary) and 40/0.08 (Large) that claims complete root canal preparation with only one instrument with adequate size and taper. The files are made of M-Wire, an innovative thermal treatment processed NiTi alloy. A special automated device is required for the reciprocal motion [29]. 

To date no study assessed the amount of DE during preparation of severely curved canals using the full-sequence rotary instruments and reciprocating systems in multi-rooted posterior teeth. The present study aimed to compare the ProTaper and WaveOne systems regarding this particular aspect.

## Materials and Methods

The study protocol was approved by research committee, dental branch, Tehran Islamic Azad University. The study was conducted on 36 human multi-rooted mandibular first molars, extracted due to periodontal problems that were without root caries, vertical or horizontal fractures, cracks (evaluated by 2.5× magnifier) and had mature apices were selected. The teeth were disinfected and root surfaces were cleaned of debris and soft tissue remnants with a periodontal curette. Initial buccolingual radiographies were taken, and teeth with internal or external resorption and previous root canal treatment or calcifications were excluded. 

The coronal access cavity was prepared using diamond burs and apical patency for each canal was confirmed with a size 10 K-file (Dentsply Maillefer, Ballaigues, Switzerland). The apical root canal width was controlled with a #15 K-file (Dentsply Maillefer, Ballaigues, Switzerland); teeth with an apical width larger than #15 were excluded. 

**Table 1.** Mean (SD) weight and Coefficient of Variation (CV) of apically extruded debris in study groups (*n*=18

**Group **	**Mean (SD) of weight (µgr)**	**CV**
**ProTaper **	35.67×10^-4^ (13.14×10^-4^)	37
**WaveOne **	45×10^-4^ (15.06×10^-4^)	34

For working length determination, a #10 K-file was inserted into the canal until the tip was slightly visible at the apical foramen. The working lengths (WL) were set at 1 mm short of the file penetration length, when the file tip was just visible at the apex. Buccolingual and mesiodistal radiographies were taken. Schneider’s technique [30] was used to determine the curvatures of the mesiobuccal (MB) canals. Teeth with curvatures between 25-35^°^ in the MB canal were selected. The crowns were adjusted so all the teeth had similar initial lengths. Each tooth was cut in half buccolingually at the furcation area, and the mesial half of the tooth was separated and randomly assigned to two groups for instrumentation (*n*=18). 

The method suggested by Myers and Montgomery [31] was modified for debris collection without the simulation of the periapical tissue resistance. The Eppendorf tubes were weighed with an electronic balance (Sartorius Cubis, Göttingen, Germany) with an accuracy of 10^−4 ^gr. Two operators took three consecutive measurements separately, and the average measurement for each tube was calculated. Stoppers were separated from Eppendorf tubes and holes were created in these stoppers to place the teeth into the tubes. Each tooth was inserted up to the cemento enamel junction through the caps, and then fixed with cyanoacrylate glue to prevent leakage of irrigating solution through the hole. A needle was placed alongside the stoppers to balance the internal and external air pressures. Then canal preparation was done using one of the following rotary file systems. 

In the first group, the root canals were prepared with ProTaper instruments (Dentsply Maillefer, Ballaigues, Switzerland) installed on a torque-controlled motor (X-Smart plus endodontic motor, Dentsply Maillefer, Ballaigues, Switzerland) at 300 rpm and a torque of 2 Ncm for 10 sec for each file. The files SX, S1, S2, F1 and F2 were applied as per manufacturer’s instruction. The root canals were irrigated with 1 mL of double-distilled water (ddH_2_O) after each instrument using a 28-gauge side end needle (Max I probe, Tulsa Dental, Dentsply, Tulsa, OK, USA). Slow speed suction was used to remove the overflowed irrigating solution from the tooth crown. The canal patency was checked with a #10 K-file. After instrumentation, 1 mL ddH_2_O was used as a final rinse. 

In the second group, the root canals were instrumented using the Primary (25.08) WaveOne reciprocating single-file (Dentsply Maillefer, Ballaigues, Switzerland) with gentle in-and-out pecking motion with short 3-mm amplitude strokes, using the same motor set on reciprocating motion. These steps were repeated three times until the working length was achieved. The root canals were irrigated with 1 mL of ddH_2_O after every three strokes in the same manner as the other group and the canal patency was checked with a # 10 K-file. 

**Table 2:** Mean (SD) preparation time and Coefficient of Variation (CV) in study groups (*n*=18

**Group **	**Mean (SD) of time (s)**	**CV**
**ProTaper **	286 (30)	11
**WaveOne **	119 (32)	19

One operator completed all root canal preparations in both groups according to the manufacturers’ suggestions. To prevent bias, an aluminum shield was used so that the operator was not able to see the root during the procedure. The same volume of irrigant was used in each root in both groups. The needle penetration depth was 2 mm shorter than the file penetration and 3 mm shorter than the WL at the apical part.

Once the instrumentation was finished, each root canal was irrigated with 2 mL of ddH_2_O, and each tooth was then removed from the Eppendorf tube. The root surface was washed with 1 mL ddH_2_O into the Eppendorf tube to collect the debris adhering to the root surface. The Eppendorf tubes were then stored in an incubator at 70^°^C for 5 days to evaporate the ddH_2_O. All of the tubes were weighted 3 more times by two operators separately, and subtracting the pre- and post-weights of the tubes determined the net weight of the apically extruded debris. 

An assistant recorded the preparation time from the initial file to the final irrigation and the overall operation time was calculated for each group in seconds. 

The data were statistically analyzed using the t-test at 95% Coefficient of Variation (CV). All Statistical analysis was performed using SPSS software (SPSS version 20.0, SPSS, Chicago, IL, USA).

## Results

Both group showed apical extrusion of debris to some extent. The amount of DE in ProTaper and WaveOne group were 35.67×10^-4^±13.14×10^-4^ and 45×10^-4^±15.06×10^-4^ gr, respectively which was 9.33 unit or 26% more in the latter group. Statistical analyses with t-test indicated that these values were significantly different (*P*<0.05). Meanwhile, the CV was in the same range for both groups (Table 1). Table 2 shows the preparation time for both groups. The canal preparation time in WaveOne group was significantly less than ProTaper group.

## Discussion

This *in vitro* study compared the amount of apical DE after root canal preparation using ProTaper rotary and WaveOne reciprocation systems. Apically extrusion of intra-canal debris and irrigants, is a common occurrence during root canal treatment, and no instrument or technique has thoroughly eliminated this problem [9]. Some studies have shown that the different techniques, motions and systems, result in different amount of DE [9, 10]. The present study showed that WaveOne extruded more debris compared to ProTaper files (26% more). Since the anatomy of the root canal system plays an important role in the overall outcome of the treatment [32], the evaluation of the extruded debris in severe curved canals in multi-rooted teeth seemed inevitable. In theory single-file reciprocating systems cut significant amount of dentin in relatively shorter time, and result in forcing debris and irrigants through the apex [19].

As demonstrated in previous studies, application of double distilled water (ddH_2_O) as an irrigant has an advantage over NaOCl as it avoids the formation of crystals [4, 24]. 

It is generally accepted and proven that hand instrumentation extrudes more debris from the apical foramen [15, 19] and crown-down technique is favorable over step-back in this regard [25, 33]. The results from the existing studies were inconclusive in regard to which engine-driven system pushes less debris in the apical direction. While some indicated that rotational movement extrude lesser amount of debris [5, 22], others demonstrated higher amount of DE in comparison to reciprocation motion [1, 4, 15]. 

The results of the present study demonstrated that the ProTaper system with full-sequence rotary motion caused less DE from the apical foramen compared to WaveOne system with the reciprocating motion. It has been discussed in previous studies that the difference between the amounts of debris extruded in these two different systems is due to the difference in the number of files and the kinematics of the motions [19]. However, based on the findings of the current study it cannot be defined which factor had a more significant effect on DE and a separate study specifically designed for this comparison is required. 

It should be mentioned that the present *in vitro* study could not reproduce the exact structure and condition of the tissues as well as pulpal status, and there were no periapical tissues that may act as a natural barrier against apical extrusion. However, the methodology used here has received the most attention and has been adopted by most studies pertaining to apical extrusion of debris [9, 24]. Regarding the number of files, single-file canal preparation in WaveOne system is expected to offer reduced working time and this fact was proved in the present study. The same result was reported in previous studies [3, 22].

## Conclusion

Within the limitations of the present study, both systems extruded debris beyond the apical foramen. Although the working time was less for the WaveOne group, it was associated with more debris extrusion than the ProTaper group. 
